# Customized modeling to predict the use of vasopressors in ICUs

**DOI:** 10.1186/cc10872

**Published:** 2012-03-20

**Authors:** A Fialho, F Cismondi, S Vieira, S Reti, L Celi, M Howell, J Sousa, S Finkelstein

**Affiliations:** 1 Massachusetts Institute of Technology, Cambridge, MA, USA; 2 Technical University of Lisbon, Instituto Superior Técnico, Lisbon, Portugal; 3 Beth Israel Deaconess Medical Centre, Boston, MA, USA

## Introduction

Vasopressors belong to a powerful class of drugs extremely useful for managing hypotension in patients with systemic shock. Being able to predict a patient's impending use of vasopressors could be beneficial as the central line insertion protocol could be initiated in a safe and timely fashion and, a central line would only be inserted if the patient has a likely future vasopressor need. Our goal in this work was to develop predictive risk models for the impending use of vasopressors in an ICU, and to make model comparisons between the general population and patients with pneumonia and pancreatitis.

## Methods

We performed a retrospective cohort study using data from four different adult ICUs at a tertiary-care hospital. Data contained 1,484 adult ICU patients, including a subgroup of 475 patients with an ICD9 diagnosis of pneumonia and 104 with an ICD9 diagnosis of pancreatitis. Two modeling approaches were used - fuzzy modeling (FM) and logistic regression (LR) - combined with a sequential forward feature selection process. For each group of patients, the selected dataset was divided into two parts: one for feature selection and the other for 10-fold cross-validation. The models' calibration was assessed using the Hosmer-Lemeshow goodness-of-fit test, and discrimination using the area under the receiver-operating curve (AUC).

## Results

All models presented good fit (*P *> 0.05) and discrimination. An AUC of 0.83 and 0.86 was obtained for the pneumonia and pancreatitis subgroups, respectively, compared to an AUC of 0.81 obtained for the general population of patients. A set of common predictive variables was found for the general population of patients: arterial base excess, noninvasive blood pressure and lactic acid. Additionally, group-specific predictive variables were found for each of the two subgroups of patients: white blood cell count for pneumonia patients, and temperature for pancreatitis patients.

## Conclusion

Generally, accurate and well-calibrated predictive risk models were obtained for the impending use of vasopressors in an ICU. However, significantly more accurate and well-calibrated models were developed for the two subpopulations - pneumonia and pancreatitis - than for the general population of ICU patients. This finding challenges one-model-fits-all approaches to overall predictive risk modeling and instead supports tailored modeling that is at least stratified at a disease level.

